# Robot-Assisted Laparoscopic Transvesical Closure of a Vesicovaginal Fistula: First Report in a Teenage Girl

**DOI:** 10.1055/a-2933-1909

**Published:** 2026-08-17

**Authors:** Pauline Lopez, Alexis Belgacem, Jenna Houari, Xavier Plainard, Aurélien Descazeaud, Quentin Ballouhey

**Affiliations:** 1 Service de Chirurgie Viscérale PédiatriqueDepartment of Pediatric SurgeryCentre Hospitalier Universitaire de LimogesLimogesAquitaine-Limousin-Poitou-CharentesFrance; 2 Service d’urologieCentre Hospitalier Universitaire de LimogesLimogesAquitaine-Limousin-Poitou-CharentesFrance

**Keywords:** vesicovaginal fistula, children, robotics

## Abstract

Vesicovaginal fistula (VVF) is rare in children and is most commonly associated with retained vaginal foreign bodies. Surgical repair is technically demanding and carries a significant risk of recurrence, which has limited the application of minimally invasive approaches. We report the first pediatric case of robot-assisted laparoscopic transvesical repair of a VVF.

A 14-year-old girl presented with longstanding urinary incontinence, malodorous vaginal discharge, and recurrent urinary tract infections, since early childhood. Initial imaging revealed a 30-mm bladder stone. Cystoscopy identified a large infratrigonal VVF associated with the stone, which had formed around a retained plastic ring. The foreign body and stone were removed through a suprapubic cystotomy. As spontaneous fistula closure did not occur after 1 year, definitive repair was undertaken. A robot-assisted laparoscopic transvesical approach was performed following cystoscopic identification of the ureteral orifices and placement of bilateral double-J stents. The fistula was closed in two watertight layers, with separate closure of the vaginal and bladder defects using absorbable sutures ( Video 1 ).

No intraoperative or postoperative complications occurred. The patient was discharged on postoperative day 2. At 1-year follow-up, she remained asymptomatic, with complete fistula closure, normal urinary continence, and normal uroflowmetry findings. Robot-assisted transvesical repair provides excellent visualization and precise suturing within the confined pelvic space, representing a promising minimally invasive option for the management of complex pediatric VVF.

## Introduction

The surgical management of vesicovaginal fistula (VVF) has been extensively described in adults, predominantly in oncologic and obstetric settings. Large case series have established the effectiveness of open transvesical repair, followed by the development of minimally invasive techniques, including laparoscopic transperitoneal repair or pneumovesicoscopy. 1 More recently, the advent of robotic surgery has further advanced the field, promoting transvaginal and transperitoneal approaches, including the recently described standardized 12-step robotic technique. 2

In contrast, VVF is rare in the pediatric population, and experience with minimally invasive repair remains limited. Since the first pediatric report in 1964, surgical management has relied predominantly on open transvesical reconstruction. 3 Although open repair provides excellent outcomes, particularly for complex fistulas, the deep pelvic location of these lesions makes surgical exposure technically demanding, especially for fistulas involving the bladder neck. While laparoscopic transperitoneal approaches may improve visualization, they may still require complementary perineal access. Moreover, vaginal or vulvar incisions are generally avoided in children because of their potential long-term morbidity. Herein, we present a didactic video demonstrating a fully robotic, single-stage transvesical repair of a VVF in a 14-year-old girl.

## Case Presentation

**Video 1** Didactic video of the patient highlighting the robotic surgical procedure, complemented by postoperative follow-up.


**Fig. 1:**
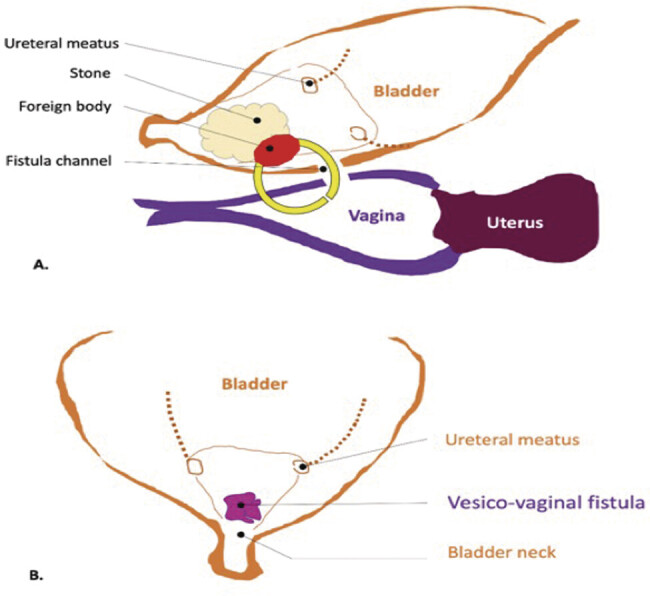
Anatomical schematic of the vesicovaginal fistula. ( **A** ) Lateral schematic view illustrating the anatomical relationship of the vesicovaginal fistula. ( **B** ) Anterior schematic view showing the location of the fistulous opening within the bladder.

A 14-year-old girl was referred for definitive management of a VVF ( Video 1 ) secondary to prolonged retention of a vaginal foreign body (plastic ring). The initial procedure consisted of cystoscopic diagnosis of the fistula ( Fig. 1 ), followed by removal of the foreign body and associated bladder stone through a limited open suprapubic cystotomy. One year later, definitive fistula repair was undertaken.

**Fig. 2:**
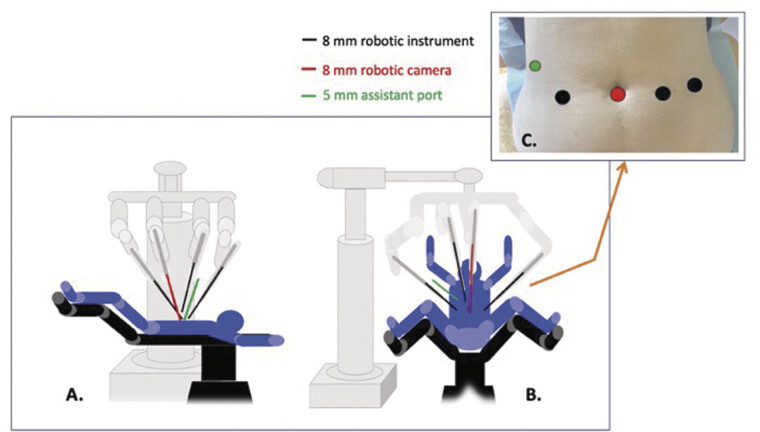
Schematic view of patient positioning, trocar placement, and robotic system positioning during robotic vesicovaginal fistula repair. ( **A** ) Lateral view, ( **B** ) front view, ( **C** ) trocar placement.

Robot-assisted laparoscopic repair was performed using four robotic ports and one assistant port. The procedure began with cystoscopic identification of the ureteral orifices, followed by bilateral placement of double-J (JJ) ureteral stents. The patient was then positioned in Trendelenburg position to facilitate a transperitoneal approach while preserving the option of perineal access if required ( Fig. 2 ). A transvesical midline cystotomy was subsequently performed, allowing meticulous dissection and complete skeletonization of the fistulous tract. After precise identification of the fistula, the vaginal and bladder defects were closed separately in two watertight layers. An omental flap was interposed between the two suture lines to reinforce the repair and minimize the risk of recurrence.

The accompanying video illustrates the endoscopic findings and provides a detailed, step-by-step demonstration of the surgical technique, highlighting the key technical principles required for safe and effective fistula repair.

No intraoperative or postoperative complications occurred. The patient was discharged on postoperative day 2. The transurethral catheter was maintained until postoperative day 22 and removed after cystography confirmed the absence of urinary leakage. The JJ stents were removed cystoscopically on postoperative day 34, confirming complete fistula closure. At 18 months of follow-up, the patient remained asymptomatic, with normal urinary continence and uroflowmetry findings.

## Discussion

VVF is exceptionally rare in the pediatric population, whether congenital or acquired. In developed countries, most pediatric cases are associated with retained vaginal foreign bodies. These fistulas are frequently located in the infratrigonal region, a location traditionally considered more challenging to repair and historically associated with less favorable surgical outcomes. 1

In the present case, management followed a planned two-stage strategy. At the initial intervention, the objective was not definitive fistula repair but removal of the retained foreign body and associated bladder stone, restoration of urinary drainage, and treatment of the local inflammatory process. Because spontaneous fistula closure remained a possibility after elimination of the underlying cause, immediate reconstruction was intentionally deferred. Delayed repair allowed complete resolution of inflammation, tissue healing, and optimization of local conditions before definitive reconstruction. This strategy also enabled careful planning of the surgical approach, with the goal of maximizing pelvic exposure while avoiding a perineal incision whenever possible.

The choice of surgical approach is primarily determined by fistula location. In adults, infratrigonal fistulas are generally approached transvaginally, whereas supratrigonal fistulas are more commonly repaired through a transvesical route. Extravesical approaches may further reduce postoperative morbidity by avoiding a large cystotomy. In children, however, published experience remains limited. Reported abdominal approaches aim to preserve the perineum, although a combined vaginal approach may still be required for infratrigonal fistulas. 2 To our knowledge, this is the first reported pediatric case of an infratrigonal VVF successfully repaired robotically through a transvesical approach without any perineal incision.

Interposition of an omental flap remains the most widely adopted method for reducing the risk of fistula recurrence. 1 Although its benefit is well established in irradiated tissues, its routine use in nonirradiated patients remains controversial, and no clear consensus currently exists. 4

Robot-assisted reconstructive urological surgery has become increasingly established in pediatric practice. 5 To our knowledge, this represents the first reported robot-assisted repair of a pediatric VVF. The robotic platform facilitated meticulous dissection and tension-free multilayer closure while avoiding a perineal incision, even for an infratrigonal fistula. Although additional experience and longer-term follow-up are needed, this case suggests that robot-assisted transvesical repair is a safe and feasible minimally invasive alternative for selected pediatric patients with VVF.

## Notes

Importance for the Pediatric Surgeon Pediatric vesicovaginal fistula (VVF) is a very rare condition, most commonly caused by vaginal foreign bodies in developed countries. Minimally invasive repair using a robotic platform is a valuable therapeutic option that should be considered in selected cases, including infratrigonal fistulas.
